# Emotion and peer problems in autistic adolescents: The role of puberty, school adjustment and bullying

**DOI:** 10.1002/jcv2.12305

**Published:** 2025-01-25

**Authors:** Erin O. Dawe‐Lane, Rob Saunders, Eirini Flouri, William P. L. Mandy

**Affiliations:** ^1^ Clinical, Educational & Health Psychology Psychology and Language Sciences University College London London UK; ^2^ Institute of Education, Psychology & Human Development University College London London UK

**Keywords:** autism spectrum disorder, bullying, emotion problems, internalising symptoms, peer problems, puberty

## Abstract

**Background:**

Emotion and peer problems tend to increase in autistic young people during adolescence. However, the extent to which endogenous (e.g., pubertal maturation) and exogenous (e.g., school adjustment, bullying) factors contribute to trajectories of emotion and peer problems in autistic young people is unclear.

**Methods:**

Using data from the Millennium Cohort Study (MCS), we fitted latent growth curves to model initial levels and growth in emotion and peer problems during adolescence. We used generalised structural equation models to investigate whether pubertal maturation, school adjustment, bullying, and timing of autism diagnosis (none, early [<8 years] or late [≥8 years]) predict initial levels and growth of emotion and peer problems in autistic and non‐autistic young people, separately for males (*n* = 780) and females (*n* = 172).

**Results:**

In females, there were significant interactions between timing of diagnosis and (a) school adjustment and (b) bullying. In females with a late diagnosis of autism, lower school adjustment and greater bullying were associated with greater growth of emotion problems during adolescence. Furthermore, in females with an early diagnosis, lower school adjustment was associated with greater initial levels of peer problems. In males, later pubertal maturation was associated with greater growth of emotion and peer problems during adolescence, irrespective of autism diagnosis. In males with an early diagnosis of autism, greater bullying was associated with greater growth of emotion problems during adolescence.

**Conclusion:**

School adjustment and bullying were associated with increasing emotion and peer problems in autistic adolescents, but their relative contribution varied according to timing of autism diagnosis and sex. Overall, this study supports the need for early identification and intervention for young autistic people experiencing poor school adjustment and bullying during adolescence.

**Key points**

- Emotion and peer problems increase in young autistic people during adolescence; however, it is unclear what factors contribute to this trend.
- We examined endogenous (e.g., puberty) and exogenous (e.g., school adjustment, bullying) factors that may contribute to emotion and peer problems in autistic and non‐autistic adolescents.
- School adjustment and bullying were associated with emotion and peer problems across adolescence in autistic young people, but their relative contribution varied according to timing of diagnosis and sex.
- The findings support early identification and intervention for autistic adolescents experiencing poor school adjustment and bullying.

## INTRODUCTION

### Autism Spectrum Disorder

Autism Spectrum Disorder (hereafter ‘autism’) is a heterogenous, neurodevelopmental condition characterised by difficulties with social relationships and communication, restricted and repetitive behaviours and atypical sensory processing (American Psychiatric Association, 2013). It is thought that between 1% and 2% of the population are autistic (Baird et al., 2006), with symptoms typically emerging in early childhood (although some cases may not be obvious until later) and persisting across the lifespan. It is purported that autistic young people can find developmental transitions challenging (Crane et al., 2021), which may have significant impacts on their emotional and social wellbeing. In this study, we examine the role of an important developmental transition, the shift from childhood to adolescence, on the trajectories of emotion and peer problems in autistic and non‐autistic young people.

The shift from childhood to adolescence is challenging for all young people (Lounds Taylor et al., 2017; Picci & Scherf, 2015; White et al., 2009) due to the convergence of pubertal maturation, the transition from primary to secondary school and increasing social complexity (e.g., choosing and maintaining friendships, gaining autonomy from parents, and discovering romantic and/or sexual relationships). In both autistic (Lai et al., 2019; White et al., 2009) and non‐autistic (Graber & Sontag, 2009) populations, internalising symptoms often emerge and increase during late childhood and adolescence. It is plausible that autistic people have specific vulnerabilities, in addition to the challenges faced by their non‐autistic peers, which contribute to increasing emotion and peer problems during adolescence. Understanding the extent to which endogenous (e.g., pubertal onset) and exogenous (e.g., school adjustment and bullying) factors influence the trajectories of emotion and peer problems in autistic young people will be important for the early identification of vulnerable individuals and the development of effective, evidence‐based intervention strategies to support the emotional and social wellbeing of autistic young people.

### Puberty

The onset of puberty signifies the biological maturation of sexual systems, which results in significant changes to morphology, cognition, emotion regulation and physiological stress responsivity (Corbett et al., 2020). The hormonal changes linked with puberty increase the risk of developing emotional and behavioural problems (Rudolph, 2014), but it is thought that the gap between physical and psychological maturity that occurs when individuals complete puberty early may increase vulnerability to deleterious mental health outcomes (Ge et al., 2011; Temelturk et al., 2021) Although this is supported by findings in females (Barendse et al., 2021; Keenan et al., 2014; Lewis et al., 2018), findings in males are conflicting; with studies suggesting that for males, both early and late pubertal maturation are associated with poor mental health outcomes (Conley & Rudolph, 2009; Mendle & Ferrero, 2012; Ullsperger & Nikolas, 2017). As such, it has been recommended that research focus on pubertal maturation in autistic young people, with the assumption that those who undergo early pubertal maturation may be at greater risk of internalising symptoms during adolescence (Gillberg & Schaumann, 1981). Nevertheless, the influence of timing of pubertal maturation on the trajectories of emotion and peer problems in autistic adolescents has not yet been explored.

### Secondary school adjustment

In addition to pubertal maturation, there are significant environmental changes that occur during adolescence, including the transition to secondary school. In the United Kingdom (UK), most autistic children attend mainstream school, which reflects increased inclusion and support for special educational needs and disabilities (SEND) (Department for Education, 2021). The transition from primary to secondary school typically occurs when children are 11 or 12 years old and represents a major ecological shift that places additional demands on individuals (Coffey, 2013). Primary schools are smaller, and children receive most of their teaching in a familiar group of peers from a single teacher and in the same classroom. By contrast, secondary schools require greater academic and social independence from students who receive instruction from different teachers in different classrooms with changing peer groups (Mandy, Murin, Baykaner, Staunton, Hellriegel, et al., 2016).

Although the transition from primary to secondary school represents a significant challenge for all young people, it should be recognised that many of the capacities that support successful adjustment to the secondary school environment, including social communication, flexibility and self‐regulation, are often impaired in autistic young people (Carter et al., 2014). Therefore, school adjustment may represent an important factor that influences the trajectories of emotion and peer problems during adolescence. Contemporaneously, the extant literature suggests that interventions designed to support individuals with autism during the transition to secondary school may have clinical benefit in terms of reducing internalising symptoms in autistic young people (Mandy, Murin, Baykaner, Staunton, COBB, et al., 2016). Nonetheless, little is known about the influence of school adjustment on the trajectories of emotion and peer problems in autistic young people.

### Increasing social complexity and bullying

The social landscape becomes increasingly complex during adolescence (Blakemore & Mills, 2014). In adolescence, the salience of social relationships with peers begins to exceed that of relationships with parents and teachers, and this social reorientation causes an unprecedented drive for acceptance and increased sensitivity to peer evaluation and rejection (Brown, 2004). In addition to this social reorientation, peer relationships become more complex as individuals are expected to navigate diverse interactions and relationships with much less support from adults than in earlier developmental periods (Carter et al., 2014). Thus, individuals must learn and re‐adjust to manage new and increased environmental demand, and those who do not successfully navigate these changes, may be at increased risk of social exclusion and bullying.

The increasing social complexity of adolescence may make the social impairments concomitant with autism more apparent (Tantam, 2003). In fact, research indicates that autistic adolescents are at risk of developing anxiety and depression related to impairments in social functioning, which may contribute to loneliness and social withdrawal, further increasing internalising symptoms (Bellini, 2006). Furthermore, although adolescence presents more opportunities for peer interaction, peer groups are increasingly self‐selecting and typically consist of individuals with similar interests, experiences and values (Ryan, 2000), which could exacerbate the social exclusion experienced by autistic young people. Research indicates that autistic young people are more likely to experience victimisation and bullying than their non‐autistic peers (Humphrey & Symes, 2011; Kloosterman et al., 2013; Rowley et al., 2012). However, the influence of bullying on the trajectories of emotion and peer problems in autistic young people has not yet been explored.

### Other considerations

Although autism can be diagnosed from 2‐years‐old, most people are diagnosed much later (e.g., during primary school), and some are not identified until adolescence or even adulthood (Hosozawa et al., 2020). Typically, late diagnosis is defined as an individual who has not been diagnosed until primary school (Miodovnik et al., 2015) and researchers have started to explore the factors that are associated with late‐diagnosed autism (Daniels & Mandell, 2014; Shattuck et al., 2009). Recent evidence suggests that the timing of diagnosis can shape the trajectory of internalising symptoms, with late‐diagnosed children showing greater growth in emotional, behavioural and social difficulties during adolescence (Mandy et al., 2022). Arguably the timing of diagnosis may influence the impact of pubertal maturation, bullying and school adjustment on the trajectory of internalising symptoms across adolescence in autism. Moreover, it should be considered that there may be different mechanisms that contribute to increasing emotion and peer problems in males and females, as well as in autistic and non‐autistic adolescents. Therefore, it will be important to explore whether there are sex differences in the impact of pubertal timing, bullying and school adjustment on emotion and peer problems in autistic and non‐autistic young people.

### Aims

In this study, we examined the impact of endogenous (e.g., pubertal maturation) and exogenous (e.g., school adjustment, bullying) factors associated with the transition from childhood to adolescence on initial levels and trajectories of emotion and peer problems in autistic and non‐autistic people. We also explored whether the impact of these factors varies according to timing of diagnosis (none, early and late). In all analyses, and for both autistic and non‐autistic young people, we explored the patterns of association with emotion and peer problems separately for males and females.

## METHODS

### Participants

Secondary analysis was conducted on data from the Millennium Cohort Study (MCS), a large, nationally representative longitudinal study exploring the development of children from 19,243 families who were born in the United Kingdom between September 2000 and January 2002. The present study incorporates data from 5 sweeps (MCS3‐MCS7), when cohort members were around 5, 7, 11, 14, and 17 years old. We modelled trajectories of internalising symptoms across ages 11–17 years, therefore, we excluded those without a single timepoint of information on emotion and peer problems between ages 11, 14 or 17. In addition, we selected only the first‐born twin or triplet, avoiding the need to account for intra‐family variability (Nath et al., 2016). Finally, we only included information provided by the main parent, excluding partner and proxy partner responses.

### Measures

#### Timing of autism diagnosis

Parents were asked, ‘has your doctor or other health professional ever told you that your child has Autism, Asperger's syndrome or other Autistic Spectrum Disorder?’ (Yes, No) when the cohort member was approximately 5, 7, 11, and 14 years old. We derived a two‐category variable based on the age that the cohort member was first reported to have an autism diagnosis: ‘early‐diagnosis’ at age 5 or 7, ‘late‐diagnosis’ at age 11 or 14 (Mandy et al., 2022). The total sample included 497 families with cohort members that had an early [<8 years] (*n* = 242) or late [≥8 years] (*n* = 255) diagnosis of autism (Mandy et al., 2022). Using exact participant matching, the sample of individuals with a diagnosis of autism (*n* = 497) were matched to a non‐autistic sample (*n* = 455) based on sex (Male, Female), ethnicity, parent income and parent education level.

#### Pubertal maturation

Parents completed an adapted version of the Pubertal Development Scale (PDS) (Petersen et al., 1988) that indicated whether the cohort member was showing evidence of puberty at age 11. The seven items asked whether the cohort member was showing any evidence of ‘growth spurt, body hair, skin changes (e.g., spots, acne), voice change, facial hair, breast development, menstruation’, which were stratified according to sex. Each item had three possible responses that were numerically coded: (0) not yet started, (1) barely started, and (2), definitely started. The question about menstruation was coded: (1) yes and (0) no. We created a total score for level of pubertal maturation at age 11 by summing the 5 items appropriate for each sex (Petersen et al., 1988).

#### School adjustment

Cohort members were asked about their motivation, attitude and engagement at school at age 14. The items asked cohort members how often they, ‘try their best at school’, ‘find school interesting’, ‘feel unhappy at school’, ‘get tired at school’, ‘misbehave or cause trouble in lessons’. Each item had four possible responses that were numerically coded: (0) all the time, (1) most of the time, (2) some of the time, and (3) never. The Cronbach's alpha (0.749) supported the items being summed to create a total score (items 1 and 2 were reverse coded) ranging from 0 – 15. In addition, cohort members were asked about their happiness with school at age 14, which was assessed using two items exploring happiness with ‘the school you go to’ and ‘schoolwork’. The items were scored on a seven‐point scale from (0) not at all happy to (6) completely happy. Based on the results from the exploratory factor analysis (see Table S3), the three measures were used to create a ‘School Adjustment’ score ranging from 0 to 27.

#### Bullying

Cohort members were asked about their experience of bullying at age 14, which was assessed using two items, ‘how often do other children hurt you or pick on you on purpose?’ and ‘how often do other children bully you online?’. The items had six possible responses that were numerically coded: (5) most days, (4) once a week, (3) once a month, (2) every few months, (1) less often, and (0) never. Based on the results from the exploratory factor analysis (see Table S3), the two items were used to create a ‘Bullying’ score ranging from 0 to 10.

#### Emotion and peer problems

The Strengths and Difficulties Questionnaire (SDQ; Goodman, 1997; Goodman, 2001), completed by parents at ages 11, 14 and 17, was used as a validated indicator of emotional and behavioural problems in adolescence. It consists of 25 items and five sub‐scales: emotional problems, peer problems, conduct problems, hyperactivity, and prosocial behaviour. The present study uses the emotion and peer problems subscales, which are often combined as an index of internalising symptoms (Goodman, 1997). We used the subscales separately in recognition of the fact that peer problems may be linked to the social and communication difficulties associated with autism rather than, or in addition to, internalising symptoms. As aforementioned, we excluded individuals without a valid measure of emotion or peer problems at any of the three timepoints.

#### Confounders

The following variables were considered confounders and measured around age 5: ethnicity (White, Non‐white), poverty (OECD 60% median income indicator: Above, Below), parent higher education (NVQ4+: Yes, No), and child cognitive ability (IQ; Flouri et al., 2018).

### Statistical analysis

All analyses were performed in STATA 17 (Stata Corporation, College Station, TX; 2021). As discussed, exploratory factor analysis (see Table S3) was used to examine the structure of self‐report items relating to school and social function at age 14, and the two derived scores for ‘School Adjustment’ and ‘Bullying’ were used in our analysis. Latent growth curve modelling (Jung & Wickrama, 2008) was used to identify the slopes and intercepts of (a) emotion and (b) peer problems from 11 to 17 years (see Table S4). For emotion and peer problems, we used the individual predicted values of the intercept (set at baseline) and slope (rate of change) in the analysis. We used the predict command in STATA to generate predicted values for the out‐of‐sample cases (e.g., the cases that were not included in the original estimation). This command employs the maximum likelihood with missing values (MLMV) estimator for those who had data on at least one time‐point, which allowed us to estimate the intercept and slope values for the full analytic sample (*n* = 952).

Missingness ranged from 0% (sex) to 28.6% (school adjustment), and missing data were imputed (30 imputed datasets) using multiple imputation by chained equations (MICE; White et al., 2011). We used all model variables to predict missing values and the MCS sampling stratum was controlled to account for the study design. We fitted two sets of generalised structural equation models (GSEM) predicting slopes and intercepts for emotion and peer problems, in both complete and imputed cases and separately for males and females. Model 1 included pubertal maturation, school adjustment and bullying. Model 2 included the covariates measured at age five (ethnicity, parent income, parent education, and child cognitive ability [IQ]). In all models, the MCS stratum were controlled to account for the study design.

## RESULTS

As discussed, our final analytic sample (*n* = 952) included 497 (52.2%) autistic and 455 (47.8%) non‐autistic people (see Table S2 for descriptive statistics). The correlations between the variables included in the models (see Table S1) were of low to moderate.

### Models

We discuss the results of the imputed cases (Tables 1 and 2) below. The complete case analyses are presented in the Supporting Information (Tables S5 and S6).

**TABLE 1:** Generalised structural equation models of timing of diagnosis, puberty, school adjustment and bullying on emotion and peer problem slope and intercepts for females.

		Model 1			Model 2		
		B	SE	95% CI	B	SE	95% CI
*Emotion Slope*							
Timing of diagnosis							
	Early	−0.274	0.418	−1.111, 0.562	−0.248	0.407	−1.058, 0.565
	Late	0.769	0.657	−0.545, 2.084	0.636	0.606	−0.577, 1.848
Puberty		−0.058	0.041	−0.139, 0.023	−0.047	0.039	−0.125, 0.029
School adjustment		−0.003	0.013	−0.029, 0.023	−0.006	0.012	−0.029, 0.019
Bullying		−0.028	0.026	−0.079, 0.023	−0.037	0.025	−0.087, 0.013
Timing of diagnosis × puberty							
	Early	0.028	0.052	−0.076, 0.133	0.009	0.048	−0.087, 0.105
	Late	−0.048	0.026	−0.100, 0.003	−0.025	0.073	−0.172, 0.122
Timing of diagnosis × school adjustment							
	Early	−0.012	0.018	−0.048, 0.023	−0.003	0.017	−0.038, 0.031
	Late	−0.048	0.026	−0.100, 0.003	−0.049*	**0.024**	**−0.089, ‐0.007**
Timing of diagnosis × bullying							
	Early	0.075	0.051	−0.027, 0.178	0.058	0.046	−0.034, 0.151
	Late	0.011	0.053	−0.102, 0.123	0.021	0.054	−0.087, 0.128
Ethnicity					0.409*	**0.189**	**0.031, 0.786**
Education					0.013	0.143	−0.273, 0.298
Income					−0.127	0.117	−0.361, 0.107
IQ					0.001	0.004	−0.007, 0.009
*Emotion Intercept*							
Timing of diagnosis							
	Early	2.455	2.033	−1.593, 6.503	1.740	2.024	−2.289, 5.769
	Late	0.063	1.808	−3.545, 3.671	0.022	1.820	−3.621, 3.665
Puberty		0.136	0.086	−0.036, 0.308	0.088	0.086	−0.083, 0.259
School adjustment		−0.039	0.042	−0.124, 0.046	−0.024	0.043	−0.110, 0.062
Bullying		0.025	0.088	−0.149, 0.199	0.095	0.083	−0.071, 0.260
Timing of diagnosis × puberty							
	Early	0.251	0.249	−0.246, 0.749	0.321	0.228	−0.133, 0.775
	Late	−0.007	0.187	−0.382, 0.367	−0.011	0.186	−0.385, 0.363
Timing of diagnosis × school adjustment							
	Early	−0.126	0.067	−0.258, 0.007	−0.289	0.221	−0.719, 0.151
	Late	0.044	0.075	−0.105, 0.194	0.049	0.073	−0.097, 0.196
Timing of diagnosis × bullying							
	Early	−0.334	0.146	−0.823, 0.154	−0.289	0.221	−0.729, 0.151
	Late	0.269*	**0.131**	**0.001, 0.536**	0.184	0.129	−0.073, 0.441
Ethnicity					0.236	0.531	−0.823, 1.296
Education					−0.085	0.350	−0.784, 0.614
Income					0.376	0.419	−0.462, 1.214
IQ					−0.038**	**0.012**	**−0.062, ‐0.015**
*Peer Slope*							
Timing of diagnosis							
	Early	0.171	0.334	−0.495, 0.839	0.101	0.321	−0.541, 0.743
	Late	0.354	0.503	−0.652, 1.359	0.234	0.747	−0.717, 1.186
Puberty		−0.021	0.026	−0.079, 0.032	−0.018	0.025	−0.068, 0.031
School adjustment		−0.006	0.008	−0.022, 0.009	−0.005	0.008	−0.021, 0.011
Bullying		−0.003	0.018	−0.039, 0.033	−0.001	0.019	−0.040, 0.037
Timing of diagnosis × puberty							
	Early	0.005	0.046	−0.086, 0.096	−0.002	0.041	−0.084, 0.079
	Late	0.019	0.051	−0.085, 0.122	0.019	0.049	−0.082, 0.119
Timing of diagnosis × school adjustment							
	Early	−0.019	0.016	−0.051, 0.013	−0.009	0.015	−0.039, 0.021
	Late	−0.038	0.022	−0.081, 0.006	−0.031	0.019	−0.071, 0.009
Timing of diagnosis × bullying							
	Early	−0.039	0.052	−0.066, 0.143	0.028	0.046	−0.064, 0.121
	Late	−0.017	0.041	−0.099, 0.064	−0.020	0.041	−0.102, 0.062
Ethnicity					0.442*	**0.192**	**0.061, 0.825**
Education					−0.003	0.115	−0.232, 0.225
Income					−0.037	0.097	−0.231, 0.157
IQ					−0.004	0.003	−0.011, 0.003
*Peer Intercept*							
Timing of diagnosis							
	Early	1.978	1.317	−0.645, 4.597	1.836	1.174	−0.499, 4.172
	Late	3.051	1.549	−0.050, 6.153	2.999*	**1.345**	**0.309, 5.697**
Puberty		0.039	0.061	−0.082, 0.161	0.058	0.058	−0.058, 0.173
School adjustment		0.038	0.023	−0.008, 0.083	0.016	0.023	−0.029, 0.061
Bullying		0.021	0.055	−0.088, 0.130	0.055	0.065	−0.073, 0.184
Timing of diagnosis × puberty							
	Early	0.139	0.161	−0.182, 0.459	0.123	1.47	−0.170, 0.415
	Late	−0.121	0.182	−0.489, 0.248	−0.147	0.171	−0.494, 0.199
Timing of diagnosis × school adjustment							
	Early	−0.101*	**0.043**	**−0.187, ‐0.016**	−0.106*	**0.041**	**−0.188, ‐0.025**
	Late	−0.057	0.074	−0.205, 0.091	−0.051	0.063	−0.176, 0.076
Timing of diagnosis × bullying							
	Early	0.126	0.127	−0.126, 0.379	0.168	0.129	−0.088, 0.425
	Late	0.233	0.119	−0.007, 0.474	0.223	0.132	−0.043, 0.489
Ethnicity					−0.980	0.635	−2.242, 0.283
Education					0.162	0.374	−0.587, 0.912
Income					−0.549	0.319	−1.193, 0.093
IQ					−0.015	0.012	−0.039, 0.009

*Note*: *N* = 172. Model 1 = Pubertal Maturation, School Adjustment, Bullying, Timing of Diagnosis and Interactions on Slopes and Intercepts of Emotion and Peer Problems. Model 2 = Model 1 + Ethnicity, Parent Income, Parent Education and Child IQ. Bold values indicate of a significant result.

*p* < 0.05*, *p* < 0.01**, *p* < 0.001***.

**TABLE 2:** Generalised structural equation models of timing of diagnosis, puberty, school adjustment and bullying on emotion and peer problems slope and intercepts for males.

		Model 1			Model 2		
		B	SE	95% CI	B	SE	95% CI
*Emotion Slope*							
Timing of diagnosis							
	Early	−0.183	0.316	−0.807, 0.441	−0.146	0.311	−0.759, 0.468
	Late	−0.199	0.283	−0.764, 0.364	−0.163	0.279	−0.721, 0.394
Puberty		−0.050**	**0.016**	**−0.081, ‐0.019**	−0.051**	**0.016**	**−0.083, ‐0.019**
School adjustment		−0.005	0.006	−0.017, 0.007	−0.005	0.006	−0.017, 0.007
Bullying		−0.004	0.017	−0.037, 0.029	−0.005	0.017	−0.038, 0.029
Timing of diagnosis × puberty							
	Early	0.033	0.026	−0.019, 0.085	0.031	0.026	−0.021, 0.083
	Late	0.043	0.039	−0.036, 0.121	0.041	0.037	−0.033, 0.116
Timing of diagnosis × school adjustment							
	Early	−0.008	0.013	−0.034, 0.018	−0.010	0.013	−0.035, 0.016
	Late	−0.001	0.014	−0.029, 0.027	−0.002	0.014	−0.030, 0.026
Timing of diagnosis × bullying							
	Early	0.065*	**0.033**	**0.000, 0.019**	0.067*	**0.033**	**0.002, 0.132**
	Late	0.010	0.035	−0.059, 0.079	0.013	0.034	−0.056, 0.081
Ethnicity					−0.121	0.077	−0.274, 0.031
Education					0.018	0.064	−0.109, 0.145
Income					−0.027	0.067	−0.159, 0.107
IQ					0.001	0.002	−0.002, 0.005
*Emotion Intercept*							
Timing of diagnosis							
	Early	1.178	0.987	−0.777, 3.13	0.920	0.990	−1.042, 2.882
	Late	3.255	1.680	−0.124, 6.635	2.665	1.509	−0.362, 0.5693
Puberty		0.034	0.050	−0.064, 0.132	0.001	0.051	−0.090, 0.112
School adjustment		−0.018	0.025	−0.068, 0.033	−0.015	0.025	−0.064, 0.035
Bullying		0.092	0.072	−0.050, 0.235	0.120	0.070	−0.019, 0.258
Timing of diagnosis × puberty							
	Early	0.043	0.106	−0.167, 0.252	0.067	0.106	−0.143, 0.277
	Late	−0.202	0.204	−0.607, 0.203	−0.153	0.192	−0.534, 0.227
Timing of diagnosis × school adjustment							
	Early	0.006	0.045	−0.083, 0.095	0.014	0.045	−0.075, 0.103
	Late	−0.069	0.074	−0.219, 0.080	−0.053	0.068	−0.190, 0.084
Timing of diagnosis × bullying							
	Early	−0.042	0.104	−0.247, 1.64	−0.081	0.106	−0.291, 0.129
	Late	0.017	0.200	−0.389, 0.422	−0.002	0.174	−0.354, 0.349
Ethnicity					0.323	0.296	−0.797, 0.391
Education					−0.204	0.296	−0.798, 0.390
Income					0.348	0.328	−0.311, 1.007
IQ					−0.025*	**0.009**	**−0.045, ‐0.005**
*Peer Slope*							
Timing of diagnosis							
	Early	−0.070	0.406	−0.877, 0.736	−0.076	0.403	−0.877, 0.725
	Late	0.117	0.316	−0.516, 0.749	0.108	0.306	−0.504, 0.720
Puberty		−0.028*	**0.012**	**−0.051, ‐0.005**	−0.027*	**0.013**	**−0.052, ‐0.002**
School adjustment		−0.001	0.005	−0.011, 0.009	−0.001	0.005	−0.011, 0.010
Bullying		0.001	0.015	−0.029, 0.031	0.001	0.016	−0.030, 0.033
Timing of diagnosis × puberty							
	Early	−0.007	0.036	−0.078, 0.064	−0.007	0.035	−0.076, 0.062
	Late	0.002	0.038	−0.073, 0.077	0.003	0.037	−0.071, 0.078
Timing of diagnosis × school adjustment							
	Early	−0.000	0.017	−0.034, 0.034	−0.000	0.017	−0.034, 0.034
	Late	−0.012	0.015	−0.043, 0.018	−0.012	0.015	−0.042, 0.017
Timing of diagnosis × bullying							
	Early	0.031	0.042	−0.053, 0.114	0.031	0.042	−0.052, 0.113
	Late	0.004	0.037	−0.070, 0.078	0.005	0.037	−0.068, 0.079
Ethnicity					0.034	0.068	−0.099, 0.167
Education					−0.035	0.067	−0.169, 0.098
Income					−0.050	0.071	−0.192, 0.091
IQ					−0.000	0.002	−0.005, 0.004
*Peer Intercept*							
Timing of diagnosis							
	Early	2.019*	**0.912**	**0.213, 3.825**	1.859*	**0.919**	**0.039, 3.677**
	Late	2.221	1.140	−0.049, 4.492	1.866	1.088	−0.306, 4.035
Puberty		0.059	0.050	−0.039, 0.158	0.046	0.049	−0.053, 0.144
School adjustment		−0.019	0.023	−0.066, 0.026	−0.018	0.023	−0.065, 0.028
Bullying		0.125*	**0.056**	**0.015, 0.235**	0.141*	**0.058**	**0.026, 0.255**
Timing of diagnosis × puberty							
	Early	−0.039	0.099	−0.234, 0.156	−0.007	0.035	−0.076, 0.062
	Late	−0.134	0.162	−0.455, 0.188	0.003	0.037	−0.071, 0.078
Timing of diagnosis × school adjustment							
	Early	−0.016	0.041	−0.097, 0.066	−0.010	0.042	−0.092, 0.072
	Late	−0.025	0.055	−0.135, 0.084	−0.015	0.054	−0.124, 0.093
Timing of diagnosis × bullying							
	Early	0.072	0.102	−0.130, 0.274	0.049	0.108	−0.163, 0.261
	Late	0.054	0.129	−0.202, 0.311	0.041	0.120	−0.198, 0.279
Ethnicity					0.210	0.285	−0.351, 0.772
Education					−0.065	0.274	−0.609, 0.478
Income					0.317	0.272	−0.224, 0.859
IQ					−0.014	0.008	−0.029, 0.002

*Note*: *N* = 780.

Model 1 = Pubertal Maturation, School Adjustment, Bullying, Timing of Diagnosis and Interactions on Slopes and Intercepts of Emotion and Peer Problems.

Model 2 = Model 1 + Ethnicity, Parent Income, Parent Education and Child IQ.

*p* < 0.05*, *p* < 0.01**, *p* < 0.001***.

In the female sample (Table 1), there was a significant positive association between the timing of diagnosis and the intercept of peer problems, such that individuals with a late diagnosis of autism had a higher intercept of peer problems. Moreover, there was a significant negative association between the timing of diagnosis and school adjustment interaction and the (a) slope of emotion problems and (b) intercept of peer problems; individuals with a late diagnosis of autism and higher school adjustment had lower slopes of emotion problems, and individuals with an early diagnosis of autism and lower school adjustment had higher intercepts of peer problems, than individuals with no diagnosis. Furthermore, there was a positive association between the timing of diagnosis and bullying interaction and the intercept of emotion problems (Model 1); individuals with a late diagnosis of autism who experienced greater levels of bullying had a higher intercept of emotion problems. However, this relationship was no longer significant after adjustment for covariates (Model 2).

In addition, there was a positive association between ethnicity and the slopes of (a) emotion and (b) peer problems (Model 2), such that white females had greater slopes of emotion and peer problems than non‐white females. Finally, there was a negative association between cognitive ability (IQ) and the intercept of emotion problems (Model 2); individuals with greater cognitive ability had lower intercepts of emotion problems, irrespective of autism diagnosis.

In the male sample (Table 2), there was a positive association between timing of diagnosis and intercept of peer problems; individuals with an early diagnosis of autism had a greater intercept of peer problems than individuals with no diagnosis. Moreover, there was a significant negative association between pubertal maturation and the slope of (a) emotion and (b) peer problems; males with late onset of puberty had greater slopes of emotion and peer problems during adolescence, irrespective of autism diagnosis. Furthermore, there was a significant positive association between bullying and the intercept of peer problems, such that individuals who experienced higher levels of bullying had a greater intercept of peer problems, irrespective of autism diagnosis.

In addition, there was a positive association between the timing of diagnosis and bullying interaction and the slope of emotion problems; individuals with an early diagnosis of autism who experienced higher levels of bullying had a greater slope of emotion problems than individuals with no diagnosis. Finally, there was a negative association between IQ and intercept of emotion problems (Model 2), such that individuals with a greater IQ at age 5 had a lower intercept of emotion problems, irrespective of autism diagnosis.

## DISCUSSION

By contrasting autistic and non‐autistic young people, we hoped to learn more about the specific factors associated with the transition from childhood to adolescence that may contribute to increasing emotion and peer problems in autistic young people. The present results indicate that pubertal maturation, school adjustment and bullying influence the trajectories of emotion and peer problems in adolescence, but that the relative contribution of these factors varies according to sex and timing of diagnosis.

### Puberty

The finding that males with later pubertal maturation had greater slopes of emotion and peer problems, suggests that later pubertal maturation is a risk factor for internalising symptoms in males, regardless of autism diagnosis. Although the hormonal changes associated with puberty may generally increase the risk for developing internalising symptoms (Rudolph, 2014), previous research on the relationship between pubertal maturation and mental health in males is mixed, with some studies suggest that early or late pubertal maturation is associated with poor mental health outcomes, respectively (Conley & Rudolph, 2009; Ullsperger & Nikolas, 2017). Our findings suggest that later pubertal maturation is associated with increasing emotion and peer problems in autistic and non‐autistic males, and while it is unclear why, it should be considered that males whose pubertal maturation occurs later than their peers may struggle to adapt to the changing environmental demands during adolescence.

We found no significant interaction between timing of diagnosis and pubertal maturation on emotion and peer problems during adolescence. Previous research on timing of pubertal maturation and autism is conflicting, with studies reporting early (Corbett et al., 2020), typical (May et al., 2017) and late (Hergüner & Hergüner, 2016; Whitehouse et al., 2011) onset of puberty in autistic people. However, the relationship between the timing of diagnosis and puberty interaction and emotion and peer problems had not previously been investigated. Our study adds to extant literature with the suggestion that later pubertal maturation is associated with the growth of emotion and peer problems during adolescence in males, regardless of autism diagnosis. Moving forward, it will be important to continue to explore the relationship between puberty and emotion and peer problems in autistic and non‐autistic adolescents to gain a clear understanding of the influence of pubertal maturation on the trajectories of emotion and peer problems.

### School adjustment

The transition from primary to secondary school represents a substantial environmental shift that places additional cognitive and social demands on all young people, but there may be specific challenges for autistic people that increase emotion and peer problems during adolescence (Picci & Scherf, 2015). In the present study, we found that females with an early diagnosis of autism and lower school adjustment had higher initial levels of peer problems compared to non‐autistic females. Interestingly, females with a late diagnosis of autism and higher school adjustment had lower growth in emotion problems during adolescence than their non‐autistic peers, which suggests that greater engagement and satisfaction at school may be a protective factor against increasing emotion problems in females with late‐diagnosed autism. As there were no significant associations between school adjustment and emotion and peer problems in males (with or without an autism diagnosis), poor school adjustment may be a risk factor for emotion and peer problems specifically in autistic females.

Carter et al. (2014) suggest several characteristics that can make adjusting to secondary school challenging for autistic people. Firstly, differences in social communication (e.g., difficulties engaging in conversation, reading non‐verbal cues, reciprocity, and eye contact) may make it difficult to form meaningful friendships and integrate with peer groups. Secondly, behavioural factors (e.g., repetitive and restrictive behaviours and limited flexibility) may challenge peer interactions and make it difficult to navigate dynamic and unpredictable social environments. Furthermore, some autistic individuals exhibit behaviours that are potentially distracting or stigmatising (e.g., stimming, aggression and self‐harm) (Kanne & Mazurek, 2011), which could lead to social exclusion or bullying. In light of this, some research (Mandy, Murin, Baykaner, Staunton, COBB, et al., 2016) has explored the acceptability and effectiveness of low‐intensity interventions for reducing problem behaviours and distress in young autistic people as they transition to secondary school; finding that children who received the interventions experienced a reduction in their emotional and behavioural problems. Our study further supports the development of interventions to ease the transition to secondary school, particularly for autistic females, as this may protect against increasing emotion and peer problems during adolescence.

### Bullying

It is perhaps not surprising that males who experienced greater bullying had greater initial levels of peer problems, irrespective of autism diagnosis. Previous research suggests that adolescents who experience bullying are at greater risk of internalising symptoms (Hawker & Boulton, 2000; Reijntjes et al., 2010). Nonetheless, it should be recognised that the relationship between bullying and emotion and peer problems symptoms may be bidirectional, whereby higher levels of emotion or peer problems puts young people at risk of bullying, as these individuals may be less socially competent and find it more difficult to integrate with peers (Christina et al., 2021). The present study provides evidence that males with higher initial levels of peer problems may be at increased risk of bullying in adolescence, regardless of autism diagnosis. The lack of an additive risk of autism diagnosis was somewhat unexpected as previous research suggests that autistic people are more vulnerable to bullying during adolescence (Fink et al., 2018). That said, males with an early diagnosis of autism who experienced greater bullying had greater slopes of emotion problems compared to their non‐autistic peers during adolescence. If causal, this link suggests that bullying may be a risk factor for increasing emotion problems during adolescence in males with early‐diagnosed autism. Recent research examining the temporal association between bullying and internalising symptoms in pre‐adolescent autistic children has indicated that bullying is an antecedent to increasing internalising symptoms, but not vice versa (Rodriguez et al., 2021). However, Tipton‐Fisler et al. (2018) explored the same temporal association in autistic adolescents and found that internalising symptoms in early adolescence predicted bullying experiences in mid‐late adolescence. Our study adds further distinction, by providing evidence that bullying in early adolescence is associated with greater growth in emotional problems in males with early diagnosed autism.

While autistic people are an extremely heterogenous group, several common characteristics can make navigating the shifting social landscape during adolescence challenging. Previous research indicates that young autistic people are especially vulnerable to feelings of loneliness (Locke et al., 2010), and experience difficulties establishing and maintaining friendships (Cresswell et al., 2019), social exclusion (Dean et al., 2014; Symes & Humphrey, 2010) and bullying (Kloosterman et al., 2013; Rowley et al., 2012) during adolescence. It is understood that the social challenges concomitant with autism may contribute to increasing internalising symptoms, especially if the individual has awareness of their social difficulties or feels intrinsically ‘different’ from others (Tantam, 2003; White & Roberson‐Nay, 2009). Furthermore, Chen et al. (2021) contend that adolescents exhibit preferences for same‐neurotype (e.g., an autistic person interacting with another autistic person) rather than cross‐neurotype (e.g., an autistic person interacting with a non‐autistic person) interactions due to shared thoughts, experiences and values. This may contribute to a ‘double empathy problem’ (Milton, 2012) whereby interpersonal mismatches mean that each group lacks sufficient insight to connect with the other. This, together with the fact that internalising symptoms can lead to reduced social engagement, feelings of isolation and exclusion, may further increase vulnerability to bullying during adolescence.

### Limitations and future directions

Our novel findings notwithstanding, we must acknowledge several study limitations. Firstly, we must acquiesce that our study design does not allow us to determine causality. That said, it has allowed us to determine the temporal association between several risk factors and emotion and peer problems. Secondly, we recognise that self and parent‐reported measures are subject to several biases that may impact the validity of the findings. Thirdly, while we tried to account for numerous factors associated with the transition from childhood to adolescence, there may be other factors that directly or indirectly (by mediation) contribute to increasing emotion and peer problems during adolescence that were not explored. Fourthly, we must acknowledge the limitations of the pubertal maturation and school adjustment measures included in the MCS, as they were measured at ages 11 and 14, respectively. Moving forward, we should collect more nuanced information on pubertal maturation, including biological measures (e.g., hormone levels), across adolescence to gain a better understanding of the influence of pubertal timing on emotion and peer problems in autistic and non‐autistic adolescents. Fifthly, it would be beneficial to explore change in school engagement, attitude and satisfaction at the time of the secondary school transition (e.g., between age 11 and 12) to allow us to better assess adjustment. Finally, although this study examined the impact of pubertal maturation, school adjustment and bullying independently, it may be that these factors interact to increase risk of emotion and peer problems in adolescence. Therefore, it will be important to elucidate the nature of their interaction in relation to the development of emotion and peer problems in both autistic and non‐autistic adolescents.

### Conclusions

Our findings indicate that pubertal maturation, school adjustment and bullying contribute to emotion and peer problems in autistic adolescents, but that the influence of these factors varies according to timing of autism diagnosis and sex. If causal, these findings support the development of interventions for young autistic people experiencing poor school adjustment and bullying. Our findings provide an additional step towards understanding the factors associated with the transition from childhood to adolescence that contribute to increasing emotion and peer problems in autistic young people.

## AUTHOR CONTRIBUTIONS

**Erin O. Dawe‐Lane:** Conceptualization; formal analysis; methodology; writing—original draft. **Rob Saunders:** Data curation; methodology; supervision; writing—review and editing. **Eirini Flouri:** Conceptualization; methodology; supervision. **William P. L. Mandy:** Conceptualization; methodology; supervision; writing—review and editing.

## CONFLICT OF INTEREST STATEMENT

The authors declare no conflicts of interest.

## ETHICAL CONSIDERATIONS

Not applicable as authors performed secondary data analyses on existing, longitudinal data from the Millenium Cohort Study.

## Supporting information

Supplementary Material

## Data Availability

The data that support the findings of this study are openly available in [UK Data Service] at [https://beta.ukdataservice.ac.uk], reference number [2000031].
