# Supplementary material for: Emotion and peer problems in autistic adolescents: The role of puberty, school adjustment and bullying
Source: JCPP Adv. 2025 Jan 25;5(3):e12305. doi: 10.1002/jcv2.12305 (PMC12446692; doi:10.1002/jcv2.12305)
Supplement: Supplementary file 1 — Supplementary Material [file JCV2-5-e12305-s001.docx]

**Supporting Information**

**Table S1.** Pearson’s correlations of variables included in the generalised structural equation models.

| Variable | M (SD) | N | 1 | 2 | 3 | 4 | 5 | 6 | 7 | 8 | 9 | 10 | 11 | 12 | 13 |
| --- | --- | --- | --- | --- | --- | --- | --- | --- | --- | --- | --- | --- | --- | --- | --- |
| 1.Sex | .82 (.385) | 952 | - |  |  |  |  |  |  |  |  |  |  |  |  |
| 2.Ethnicity | .88 (.320) | 951 | -.059 | - |  |  |  |  |  |  |  |  |  |  |  |
| 3.Poverty | .46 (.499) | 910 | -.004 | -.071* | - |  |  |  |  |  |  |  |  |  |  |
| 4.Education | .33 (.472) | 763 | -.027 | -.118** | -.214** | - |  |  |  |  |  |  |  |  |  |
| 5.IQ | 96.57 (16.093) | 838 | -.043 | .153** | -.149** | .122** | - |  |  |  |  |  |  |  |  |
| 6.Timing Diagnosis | .79 (.838) | 952 | -.027 | .007 | .032 | -.094** | -.135** | - |  |  |  |  |  |  |  |
| 7.Pubertal Maturation | 2.94 (2.431) | 817 | -.507** | -.038 | .035 | -.021 | -.032 | .075* | - |  |  |  |  |  |  |
| 8.Bullying | 1.77 (2.179) | 683 | -.112** | .119** | .057 | .020 | .024 | .133** | .001 |  |  |  |  |  |  |
| 9.School Adjustment | 19.37 (5.370) | 680 | .143** | -.081* | -.110** | .138** | .105** | -.197** | -.069 | - |  |  |  |  |  |
| 10.Emotion Slope | -.2080 (.4262) | 952 | -.053 | .016 | -.055 | .029 | .060 | -.096** | -.067 | -.276** | - |  |  |  |  |
| 11.Emotion Intercept | 2.8365 (1.769) | 952 | -.097 | .023 | .113** | -.137** | -.261** | .351** | .134** | .078* | -.092* | - |  |  |  |
| 12.Peer Slope | -.0611 (.3746) | 952 | -.003 | .044 | -.029 | -.019 | .010 | -.075 | -.079* | .183** | -.212** | -.299** | - |  |  |
| 13.Peer Intercept | 2.6325 (1.674) | 952 | .020 | .007 | .058 | -.075* | -.197** | .390** | .043 | .021 | -.069 | .533** | -.060 | - |  |
|  |  |  |  |  |  |  |  |  |  | .314** | -.206** | -.143** | .573** | -.243** | - |

***Note.*** 1-5; covariates measured at 5 years (0= male, non-white, above median income, no higher education : 1=female, white, below median income, higher education), 6; timing of diagnosis measured at 5, 7, 11 and 14 (0=no diagnosis, 1=early diagnosis and 2=late diagnosis), 7; Pubertal Maturation, 8-9; School Adjustment and Bullying measured at age 14, 10-13; Strengths and Difficulties Questionnaire (SDQ) Emotion and Peer Problems slope and intercepts calculated from ages 11, 14 and 17.

**Table S2.** Descriptive statistics for the analytic sample (unadjusted).

| Variable |  | Autistic |  | Non-Autistic |  |  |
| --- | --- | --- | --- | --- | --- | --- |
|  |  | N | % | N | % |  |
| Sex |  |  |  |  |  |  |
|  | Female | 90 | 18.1 | 82 | 18.0 | X^2^ _(1)_ = .001, |
|  | Male | 407 | 81.9 | 373 | 82.0 | p=.972 |
| Ethnicity |  |  |  |  |  |  |
|  | White | 438 | 88.5 | 403 | 88.5 | X^2^ _(1)_ =.016, |
|  | Non-White | 58 | 11.5 | 52 | 11.5 | p=.898 |
| Income |  |  |  |  |  |  |
|  | Above | 246 | 54.0 | 246 | 54.0 | X^2^ _(1)_ =.000, |
|  | Below | 209 | 46.0 | 209 | 46.0 | p=.999 |
| Higher Education |  |  |  |  |  |  |
| (NVQ 4+) |  |  |  |  |  |  |
|  | Yes | 110 | 29.4 | 104 | 29.3 | X^2^ _(1)_ =.002, |
|  | No | 274 | 70.6 | 252 | 70.7 | p=.957 |

|  | Autistic |  |  | Non-Autistic |  |  |  |
| --- | --- | --- | --- | --- | --- | --- | --- |
|  | M | SE | N | M | SE | N |  |
| IQ | 93.65 | (.87) | 393 | 99.14 | (.69) | 445 | *t*_(836)_ = 4.997, p<.001 |
| Pubertal Maturation | 3.13 | (.121) | 424 | 2.74 | (.118) | 393 | *t*_(815)_ = -2.249), p=.025 |
| Bullying | 2.10 | (.133) | 321 | 1.48 | (.102) | 362 | *t*_(681)_ = -3.743, p<.001 |
| School Adjustment | 18.35 | (.330) | 319 | 20.28 | (.247) | *361* | *t*_(678)_ = 4.740, p<.001 |

**Table S3.** Exploratory Factor Analysis

|  | 1 | 2 | Communalities |
| --- | --- | --- | --- |
| Happiness with School | **.817** | -.176 | .698 |
| Happiness with School Work | **.837** | -.046 | .703 |
| School Engagement and Attitude | **.804** | -.118 | .660 |
| Bullying | -.177 | **.790** | .656 |
| Bullying Online | -.047 | **.836** | .701 |
| Eigenvalue | 2.277 | 1.141 |  |
| N | 679 | 679 |  |

***Note.*** Principal components analysis with varimax rotation

The exploratory factor analysis (principal components analysis) indicated a 2-factor solution (KMO=.711, X^2^_(10)_ =681.280, p<.001) (Table 2): (1) School Adjustment, and (2) Bullying, which explained 68.36% of the total variance. Factor 1, labelled school adjustment, included measures of happiness ‘at school’ and ‘with schoolwork’ and the total ‘school engagement and attitude’ score, and explained 45.55% of the total variance. Factor 2, labelled Bullying, included two items ‘how often do other children pick on you or hurt you on purpose’ and ‘how often do other children pick on you or hurt you on purpose online’ and explained 22.82% of the total variance. One item, ‘how many of your close friends go to the same school as you’ did not load onto either factor and was excluded from the final factor analysis. We devised total scores for (1) School Adjustment, and (2) Bullying, which we included in the final models.

**Table S4.** Latent Growth Curve Models for Emotion and Peer Problems

|  | Emotion Problem |  | Peer Problem |  |
| --- | --- | --- | --- | --- |
|  | Estimate | SE | Estimate | SE |
| Means |  |  |  |  |
| Intercept | 2.837 | .085 | 2.633 | .079 |
| Slope | -.208 | .052 | -.061 | .047 |
| Variance |  |  |  |  |
| Intercept | 4.423 | .340 | 3.882 | .289 |
| Slope | .677 | .130 | .543 | .108 |
| Covariance |  |  |  |  |
| Intercept-Slope | -.659 | .167 | -.498 | .140 |
| Residual Variance |  |  |  |  |
| Time 1 | 2.581 | .140 | 2.168 | .117 |
| Time 2 | 2.581 | .140 | 2.168 | .117 |
| Time 3 | 2.581 | .140 | 2.168 | .117 |

Note. SE = standard error.

**Table S5.** Generalised Structural Equation Models of Timing of Diagnosis, Puberty, School Adjustment and Bullying on Emotion and Peer Problems Slope and Intercepts for Females.

|  |  | Model 1 |  |  | Model 2 |  |  |
| --- | --- | --- | --- | --- | --- | --- | --- |
|  |  | B | SE | 95% CI | B | SE | 95% CI |
| Emotion Slope | | | | | | | |
| Timing of Diagnosis |  |  |  |  |  |  |  |
|  | Early | -.250 | .426 | -1.101, .600 | -.423 | .389 | -1.204, .357 |
|  | Late | 1.127 | .625 | -.122, 2.375 | **1.368*** | **.545** | **.277, 2.459** |
| Puberty |  | -.057 | .048 | -.154, .039 | -.054 | .050 | -.154, .047 |
| School Adjustment |  | -.003 | .011 | -.024, .018 | -.006 | .009 | -.026, .013 |
| Bullying |  | -.017 | .029 | -.077, .042 | -.007 | .031 | -.069, .054 |
| Timing of Diagnosis X Puberty |  |  |  |  |  |  |  |
|  | Early | .013 | .062 | -.112, .137 | .082 | .049 | -.017, .182 |
|  | Late | -.020 | .071 | -.162, .122 | -.053 | .083 | -.219, .113 |
| Timing of Diagnosis X School Adjustment |  |  |  |  |  |  |  |
|  | Early | -.005 | .019 | -.043, .034 | -.018 | .019 | -.055, .021 |
|  | Late | **-.069*** | **.027** | **-.123, -.014** | **-.058*** | **.029** | **-.115, -.000** |
| Timing of Diagnosis X Bullying |  |  |  |  |  |  |  |
|  | Early | .045 | .055 | -.065, .155 | .052 | .061 | -.069, .175 |
|  | Late | -.075 | .054 | -.182, .032 | **-.167**** | **.056** | **-.279, -.054** |
| Ethnicity |  |  |  |  | -.076 | .142 | -.360, .209 |
| Education |  |  |  |  | .112 | .186 | -.260, .484 |
| Income |  |  |  |  | .036 | .179 | -.322, .395 |
| IQ |  |  |  |  | -.002 | .006 | -.015, .011 |

|  |  | Model 1 |  |  | Model 2 |  |  |
| --- | --- | --- | --- | --- | --- | --- | --- |
|  |  | B | SE | 95% CI | B | SE | 95% CI |
| Emotion Intercept | | | | | | | |
| Timing of Diagnosis |  |  |  |  |  |  |  |
|  | Early | 2.693 | 1.972 | -1.244, 6.629 | 1.343 | 2.439 | -3.539, 6.227 |
|  | Late | -2.123 | 2.072 | -6.259, 2.012 | -1.764 | 2.587 | -6.944, 3.416 |
| Puberty |  | .090 | .091 | -.091, .271 | .035 | .094 | -.154, .224 |
| School Adjustment |  | -.043 | .041 | -.125, .039 | -.044 | .046 | -.137, .048 |
| Bullying |  | .009 | .089 | -.168, .186 | .048 | .094 | -.141, .237 |
| Timing of Diagnosis X Puberty |  |  |  |  |  |  |  |
|  | Early | .514 | .287 | -.059, 1.087 | .605 | .422 | -.241, 1.451 |
|  | Late | .064 | .219 | -.373, .500 | .036 | .276 | -.516, .589 |
| Timing of Diagnosis X School Adjustment |  |  |  |  |  |  |  |
|  | Early | **-.198**** | **.074** | **-.346, -.049** | -.159 | .102 | -.365, .045 |
|  | Late | .119 | .069 | -.021, .259 | .091 | .091 | -.091, .273 |
| Timing of Diagnosis X Bullying |  |  |  |  |  |  |  |
|  | Early | -.500 | .247 | -.992, .000 | -.362 | .326 | -1.014, .289 |
|  | Late | **.504**** | **.153** | **.199, .810** | **.393*** | **.154** | **.084, .702** |
| Ethnicity |  |  |  |  | .978 | .406 | .166, 1.780 |
| Education |  |  |  |  | -.064 | .476 | -1.017, .889 |
| Income |  |  |  |  | .144 | .621 | -1.100, 1.388 |
| IQ |  |  |  |  | **-.047*** | **.022** | **-.091, -.001** |

|  |  | Model 1 |  |  | Model 2 |  |  |
| --- | --- | --- | --- | --- | --- | --- | --- |
|  |  | B | SE | 95% CI | B | SE | 95% CI |
| Peer Slope | | | | | | | |
| Timing of Diagnosis |  |  |  |  |  |  |  |
|  | Early | .242 | .385 | -.537, 1.012 | .039 | .372 | -.706, .785 |
|  | Late | .695 | .531 | -.364, 1.755 | .739 | .505 | -.271, 1.750 |
| Puberty |  | -.014 | .030 | -.075, .046 | -.022 | .031 | -.085, .041 |
| School Adjustment |  | -.007 | .007 | -.020, .006 | -.005 | .008 | -.021, .011 |
| Bullying |  | .003 | .015 | -.028, .033 | .017 | .017 | -.016, .051 |
| Timing of Diagnosis X Puberty |  |  |  |  |  |  |  |
|  | Early | -.030 | .053 | -.136, .075 | -.015 | .068 | -.152, .122 |
|  | Late | .015 | .052 | -.089, .119 | -.021 | .057 | -.135, .093 |
| Timing of Diagnosis X School Adjustment |  |  |  |  |  |  |  |
|  | Early | -.010 | .019 | -.049, .029 | -.004 | .027 | -.058, .051 |
|  | Late | **-.056*** | **.024** | **-.103, -.009** | -.032 | .022 | -.077, .013 |
| Timing of Diagnosis X Bullying |  |  |  |  |  |  |  |
|  | Early | .022 | .044 | -.067, .110 | .027 | .071 | -.114, .169 |
|  | Late | -.075 | .047 | -.169, .019 | **-.149**** | **.048** | **-.245, -.054** |
| Ethnicity |  |  |  |  | -.009 | .111 | -.231, .213 |
| Education |  |  |  |  | .029 | .132 | -.235, .295 |
| Income |  |  |  |  | .104 | .144 | -.185, .393 |
| IQ |  |  |  |  | -.006 | .005 | -.017, .004 |

|  |  | Model 1 |  |  | Model 2 |  |  |
| --- | --- | --- | --- | --- | --- | --- | --- |
|  |  | B | SE | 95% CI | B | SE | 95% CI |
| Peer Intercept | | | | | | | |
| Timing of Diagnosis |  |  |  |  |  |  |  |
|  | Early | 1.513 | 1.386 | -1.255, 4.279 | 1.061 | 1.350 | -.1643, 3.765 |
|  | Late | 2.578 | 1.848 | -1.111, 6.266 | 2.177 | 1.959 | -1.748, 6.101 |
| Puberty |  | .032 | .067 | -.103, .166 | .048 | .079 | -.112, .207 |
| School Adjustment |  | .044 | .022 | -.001, .089 | .024 | .023 | -.021, .069 |
| Bullying |  | .023 | .064 | -.103, .150 | -.014 | .088 | -.189, .161 |
| Timing of Diagnosis X Puberty |  |  |  |  |  |  |  |
|  | Early | .187 | .161 | -.133, .508 | .284 | .206 | -.127, .696 |
|  | Late | -.106 | .165 | -.436, .224 | -.025 | .203 | -.431, .381 |
| Timing of Diagnosis X School Adjustment |  |  |  |  |  |  |  |
|  | Early | **-.109**** | **.039** | **-.187, .031** | **-.102*** | **.047** | **-.195, -.008** |
|  | Late | -.016 | .075 | -.167, .134 | -.073 | .081 | -.235, .089 |
| Timing of Diagnosis X Bullying |  |  |  |  |  |  |  |
|  | Early | .186 | .125 | -.063, .435 | .106 | .149 | -.194, .406 |
|  | Late | .159 | .147 | -.034, .552 | **.459*** | **.187** | **.086, .834** |
| Ethnicity |  |  |  |  | .242 | .505 | -.770, 1.254 |
| Education |  |  |  |  | -.249 | .477 | -1.204, .705 |
| Income |  |  |  |  | -.907 | .456 | -1.820, .005 |
| IQ |  |  |  |  | -.015 | .019 | -.054, .023 |
| **Note.**  N= 172.  Model 1 = Pubertal Maturation, School Adjustment, Bullying, Timing of Diagnosis and Interactions on Slopes and Intercepts of Emotion and Peer Problems.  Model 2 = Model 1 + Ethnicity, Parent Income, Parent Education and Child IQ.  P<.05*, p<.01**, p<.001*** | | | | | | | |

In the complete case analysis for females (Table 4), there was an association between timing of diagnosis and the slope of emotion problems (Model 1 and 2); individuals with a late diagnosis of autism had greater slopes of emotion problems during adolescence than individuals with no diagnosis. Moreover, there was an association between the timing of diagnosis and bullying interaction and the slope of (1) emotion and (2) peer problems (Table 4, Model 2). Females with a late diagnosis who experience lower levels of bullying had greater slopes of emotional problems. Females with a late diagnosis who experienced greater bullying had greater slopes of peer problems, than those with no diagnosis. Furthermore, individuals with an early diagnosis of autism who experienced greater Bullying had greater intercepts of peer problems during adolescence. Furthermore, there was an association between the timing of diagnosis and school adjustment interaction and the (1) slope of emotion problems, (2) intercept of emotion problems and (3) slope of peer problems. Females with an early diagnosis of autism and lower school adjustment had a greater intercept of emotion problems and slope of peer problems during adolescence. Moreover, females with a late diagnosis of autism and lower school adjustment had greater slope of emotion problems. Finally, there was an association between timing of diagnosis and the intercept of peer problems; females with a late diagnosis had greater intercepts of peer problems at age 11 than individuals with no diagnosis.

**Table S6.** Generalised Structural Equation Models of Timing of Diagnosis, Puberty, School Adjustment and Bullying on Emotion and Peer Problems Slope and Intercepts for Males.

|  |  | Model 1 |  |  | Model 2 |  |  |
| --- | --- | --- | --- | --- | --- | --- | --- |
|  |  | B | SE | 95% CI | B | SE | 95% CI |
| Emotion Slope | | | | | | | |
| Timing of Diagnosis |  |  |  |  |  |  |  |
|  | Early | -.365 | .379 | -1.113, .383 | -.106 | .345 | -.786, .574 |
|  | Late | -.399 | .281 | -.953, .155 | .070 | .383 | -.686, .826 |
| Puberty |  | **-.067***** | **.019** | **-.105, -.031** | **-.068***** | **.019** | **-.105, -.031** |
| School Adjustment |  | -.005 | .007 | -.018, .009 | -.005 | .008 | -.020, .009 |
| Bullying |  | -.007 | .019 | -.044, .031 | -.016 | .019 | -.054, .022 |
| Timing of Diagnosis X Puberty |  |  |  |  |  |  |  |
|  | Early | .048 | .029 | -.008, .105 | .051 | .026 | -.001, .103 |
|  | Late | .087 | .039 | .009, .165 | **.089*** | **.044** | **.002, .176** |
| Timing of Diagnosis X School Adjustment |  |  |  |  |  |  |  |
|  | Early | -.004 | .014 | -.032, .024 | -.015 | .014 | -.042, .013 |
|  | Late | .006 | .012 | -.017, .029 | -.013 | .016 | -.044, .019 |
| Timing of Diagnosis X Bullying |  |  |  |  |  |  |  |
|  | Early | **.097*** | **.042** | **.013, .180** | **.099**** | **.035** | **.031, .167** |
|  | Late | .015 | .029 | -.043, .073 | -.017 | .026 | -.069, .035 |
| Ethnicity |  |  |  |  | -.018 | .089 | -.195, .158 |
| Education |  |  |  |  | .052 | .062 | -.071, .176 |
| Income |  |  |  |  | -.004 | .075 | -.151, .145 |
| IQ |  |  |  |  | .003 | .002 | -.002, .008 |

|  |  | Model 1 |  |  | Model 2 |  |  |
| --- | --- | --- | --- | --- | --- | --- | --- |
|  |  | B | SE | 95% CI | B | SE | 95% CI |
| Emotion Intercept | | | | | | | |
| Timing of Diagnosis |  |  |  |  |  |  |  |
|  | Early | .962 | 1.039 | -1.087, 3.010 | .546 | 1.215 | -1.849, 2.942 |
|  | Late | **2.293*** | **1.015** | **.292, 4.294** | .085 | .984 | -1.856, 2.026 |
| Puberty |  | .060 | .055 | -.048, .169 | .022 | .052 | -.079, .124 |
| School Adjustment |  | -.011 | .026 | -.062, .039 | -.010 | .025 | -.058, .038 |
| Bullying |  | .084 | .068 | -.049, .218 | .125 | .065 | -.004, .254 |
| Timing of Diagnosis X Puberty |  |  |  |  |  |  |  |
|  | Early | .067 | .126 | -.181, .314 | .110 | .129 | -.145, .366 |
|  | Late | -.066 | .129 | -.322, .190 | -.082 | .118 | -.314, .150 |
| Timing of Diagnosis X School Adjustment |  |  |  |  |  |  |  |
|  | Early | .009 | .048 | -.085, .103 | .027 | .054 | -.078, .133 |
|  | Late | -.068 | .044 | -.156, .019 | .019 | .042 | -.063, .101 |
| Timing of Diagnosis X Bullying |  |  |  |  |  |  |  |
|  | Early | -.021 | .105 | -.228, .185 | -.113 | .117 | -.343, .118 |
|  | Late | .041 | .108 | -.173, .254 | .178 | .091 | -.002, .254 |
| Ethnicity |  |  |  |  | .083 | .259 | -.428, .595 |
| Education |  |  |  |  | **-.375*** | **.172** | **-.714, -.036** |
| Income |  |  |  |  | .434 | .245 | -.049, .918 |
| IQ |  |  |  |  | -.030 | .007 | -.043, -.017 |

|  |  | Model 1 |  |  | Model 2 |  |  |
| --- | --- | --- | --- | --- | --- | --- | --- |
|  |  | B | SE | 95% CI | B | SE | 95% CI |
| Peer Slope | | | | | | | |
| Timing of Diagnosis |  |  |  |  |  |  |  |
|  | Early | -.259 | .350 | -.950, .430 | -.054 | .259 | -.566, .459 |
|  | Late | .090 | .271 | -.444, .624 | .399 | .334 | -.260, 1.058 |
| Puberty |  | **-.029*** | **.012** | **-.054, -.005** | -.023 | .013 | -.048, .002 |
| School Adjustment |  | -.001 | .006 | -.013, .010 | .003 | .007 | -.009, .016 |
| Bullying |  | -.001 | .013 | -.027, .026 | -.000 | .014 | -.028, .027 |
| Timing of Diagnosis X Puberty |  |  |  |  |  |  |  |
|  | Early | -.014 | .024 | -.062, .035 | -.018 | .023 | -.063, .026 |
|  | Late | -.015 | .011 | -.036, .006 | -.021 | .014 | -.048, .007 |
| Timing of Diagnosis X School Adjustment |  |  |  |  |  |  |  |
|  | Early | -.000 | .013 | -.026, .025 | -.008 | .011 | -.030, .014 |
|  | Late | -.015 | .011 | -.036, .006 | -.021 | .014 | -.048, .007 |
| Timing of Diagnosis X Bullying |  |  |  |  |  |  |  |
|  | Early | .071 | .046 | -.019, .161 | .054 | .031 | -.006, .115 |
|  | Late | -.011 | .029 | -.069, .047 | -.056 | .029 | -.112, .000 |
| Ethnicity |  |  |  |  | .083 | .085 | -.085, .251 |
| Education |  |  |  |  | -.019 | .049 | -.116, .078 |
| Income |  |  |  |  | .021 | .052 | -.081, .123 |
| IQ |  |  |  |  | .000 | .001 | -.003, .003 |

|  |  | Model 1 |  |  | Model 2 |  |  |
| --- | --- | --- | --- | --- | --- | --- | --- |
|  |  | B | SE | 95% CI | B | SE | 95% CI |
| Peer Intercept | | | | | | | |
| Timing of Diagnosis |  |  |  |  |  |  |  |
|  | Early | **1.929**** | **.945** | **.067, 3.792** | **3.071**** | **1.007** | **1.085, 5.056** |
|  | Late | 1.297 | 1.017 | -.707, 3.300 | -.103 | 1.132 | -2.336, 1.129 |
| Puberty |  | .067 | .058 | -.047, .181 | .027 | .059 | -.089, .144 |
| School Adjustment |  | -.019 | .026 | -.070, .031 | -.013 | .026 | -.065, .038 |
| Bullying |  | .108 | .060 | -.011, .228 | **.125*** | **.058** | **.012, .239** |
| Timing of Diagnosis X Puberty |  |  |  |  |  |  |  |
|  | Early | -.053 | .110 | -.270, .164 | -.056 | .114 | -.281, .169 |
|  | Late | -.105 | .128 | -.357, .148 | .016 | .129 | -.239, .271 |
| Timing of Diagnosis X School Adjustment |  |  |  |  |  |  |  |
|  | Early | -.011 | .0436 | -.097, .075 | -.058 | .046 | -.149, .033 |
|  | Late | -.006 | .047 | -.099, .088 | .034 | .051 | -.067, .135 |
| Timing of Diagnosis X Bullying |  |  |  |  |  |  |  |
|  | Early | .165 | .086 | -.008, .337 | .136 | .099 | -.059, .331 |
|  | Late | .086 | .131 | -.171, .344 | .175 | .131 | -.082, .433 |
| Ethnicity |  |  |  |  | -.074 | .277 | -.620, .472 |
| Education |  |  |  |  | -.188 | .195 | -.574, .197 |
| Income |  |  |  |  | **.455*** | **.212** | **.037, .872** |
| IQ |  |  |  |  | **-.014*** | **.007** | **-.027, -.000** |
| **Note.**  N= 172.  Model 1 = Pubertal Maturation, School Adjustment, Bullying, Timing of Diagnosis and Interactions on Slopes and Intercepts of Emotion and Peer Problems.  Model 2 = Model 1 + Ethnicity, Parent Income, Parent Education Level and Child IQ.  P<.05*, p<.01**, p<.001*** | | | | | | | |

In the complete case analyses for Males (Table 5, Model 1), there was an association between timing of diagnosis and the intercept of (1) emotion and (2) peer problems; males with a late diagnosis of autism had higher intercepts of emotion and peer problems during adolescence than individuals with no diagnosis, but this was not robust to adjustment. In the adjusted model (Table 5, Model 2), there was a significant association between the timing of diagnosis and pubertal maturation interaction and the slope of emotion problems; males with a late diagnosis who went through early pubertal maturation had greater slopes of emotion problems during adolescence. There were negative associations between pubertal maturation and the slope of (1) emotion and (2) peer problems; males who went through pubertal late had greater growth in emotion and peer problems during adolescence, regardless of autism diagnosis. There was an association between the timing of diagnosis and bullying interactions and the slope of emotion problems; individuals with an early diagnosis who experienced greater levels of bullying had greater slopes of emotion problems during adolescence.
